# Hydroxynorketamine, but not ketamine, acts via α7 nicotinic acetylcholine receptor to control presynaptic function and gene expression

**DOI:** 10.1038/s41398-024-02744-y

**Published:** 2024-01-22

**Authors:** Debarpan Guhathakurta, Aneta Petrušková, Jan Yağız Buchholz, Bartomeu Perelló-Amorós, Renato Frischknecht, Daniela Anni, Eva-Maria Weiss, Martin Walter, Anna Fejtová

**Affiliations:** 1 Department of Psychiatry and Psychotherapy, Universitätsklinikum Erlangen, Friedrich-Alexander-Universität Erlangen-Nürnberg, Erlangen, Germany; 2 National Institute of Mental Health, Klecany, Czech Republic; 3 Third Faculty of Medicine, Charles University, Prague, Czech Republic; 4 Department of Biology, Animal Physiology, Friedrich-Alexander-Universität Erlangen-Nürnberg, Erlangen, Germany; 5 Department of Psychiatry and Psychotherapy, Universitätsklinikum Jena, Jena, Germany

**Keywords:** Molecular neuroscience, Depression

## Abstract

Ketamine is clinically used fast-acting antidepressant. Its metabolite hydroxynorketamine (HNK) shows a robust antidepressant effect in animal studies. It is unclear, how these chemically distinct compounds converge on similar neuronal effects. While KET acts mostly as N-methyl-d-aspartate receptor (NMDAR) antagonist, the molecular target of HNK remains enigmatic. Here, we show that KET and HNK converge on rapid inhibition of glutamate release by reducing the release competence of synaptic vesicles and induce nuclear translocation of pCREB that controls expression of neuroplasticity genes connected to KET- and HNK-mediated antidepressant action. Ro25-6981, a selective antagonist of GluN2B, mimics effect of KET indicating that GluN2B-containing NMDAR might mediate the presynaptic effect of KET. Selective antagonist of α7 nicotinic acetylcholine receptors (α7nAChRs) or genetic deletion of *Chrna7*, its pore-forming subunit, fully abolishes HNK-induced synaptic and nuclear regulations, but leaves KET-dependent cellular effects unaffected. Thus, KET or HNK-induced modulation of synaptic transmission and nuclear translocation of pCREB can be mediated by selective signaling via NMDAR or α7nAChRs, respectively. Due to the rapid metabolism of KET to HNK, it is conceivable that subsequent modulation of glutamatergic and cholinergic neurotransmission affects circuits in a cell-type-specific manner and contributes to the therapeutic potency of KET. This finding promotes further exploration of new combined medications for mood disorders.

## Introduction

Major depressive disorder (MDD) is a psychiatric disorder affecting 280 million people globally [1]. Current pharmacological treatments, targeting prevalently the monoaminergic systems, need to be taken for weeks to obtain antidepressant action, cause considerable side effects and leave substantial proportion of patients unresponsive to treatment [2, 3]. Since the 1990s, the glutamatergic system has been implicated in the etiology and neurobiology of MDD, leading to research exploring the efficacy of novel therapeutics that target this system [4–6]. Ketamine (KET) showed robust, rapid, and sustained antidepressant effects in preclinical and clinical studies [7–9]. Recently, FDA approved Esketamine for treatment-resistant MDD [10]. However, adverse effects of KET including dissociation, psychotomimetic effects, cognitive impairment and abuse liability prevent its broad application for MDD treatment [11–13]. Therefore, significant efforts are being made to dissect the mechanism of its action, in order to develop compounds with higher safety and adherence.

KET acts as a non-competitive antagonist of N-methyl-d-aspartate receptors (NMDARs), which is crucial for its biological effects as an antidepressant [14]. It has been proposed that transient inhibition of NMDAR-dependent synaptic transmission mediated by KET leads, as assessed 1 day later, to sustained potentiation of glutamatergic neurotransmission [15]. This is in line with studies that demonstrated an acute decline in evoked glutamate release in mice prefrontal cortex (PFC) and subiculum [16, 17], and an inverse correlation between sustained antidepressant effects of KET and tissue glutamine levels in the mPFC of depressed patients [18]. KET is rapidly metabolized to hydroxynorketamine (HNK), which can be detected in human plasma, rodent brain and plasma, few minutes after KET administration [19–21]. The administration of KET variant that can’t be metabolized attenuated KET-induced antidepressant-like effects in rodents, indicating that some biological effects of KET might be mediated by its metabolite HNK [20–22]. Indeed, a direct administration of HNK reduced depressive behavior [23–28] and protected against stress-induced behavior in rodents [29]. These findings are important as HNK lack the psychotomimetic effects and abuse potential of its parent compound KET [20, 30–32]. Indeed, mood improvement but weaker dissociation was observed upon oral administration of KET formulation with delayed absorption that results in reduced KET blood concentration, but build-up of its longer-living metabolites [33, 34]. Thus, understanding HNK-induced signaling is of utmost importance.

There is a clear consensus that both KET and HNK induce signaling leading to a reconfiguration of expression of neuronal proteins. The signaling cascades implicated in maintaining the antidepressant effects of KET downstream of NMDARs include the mitogen-activated protein kinase/extracellular signal-regulated kinase (MAPK/ERK), which controls expressional regulation of neuroplasticity genes and mammalian target of rapamycin (mTOR), the major checkpoint of local protein synthesis [35, 36]. Both KET and HNK upregulate glutamatergic neurotransmission via increased expression of post-synaptic α-amino-3-hydroxy-5-methyl-4-isoxazolepropionic acid receptors (AMPARs) and elevate hippocampal BDNF levels 24 h after treatment [20, 37–39]. The immediate effects of KET and HNK are currently a matter of intense investigation. Several recent studies report an acute inhibition of glutamate release upon KET and HNK application [16, 17, 40]. These effects rely on the inhibition of synaptic neurotransmitter release implicating regulation of presynaptic function as an important mechanism underlying fast antidepressant action of KET and HNK [17].

Despite the similar effects and overlapping signaling elicited by KET and HNK, recent studies reported that some biological effects differ between KET and HNK [41, 42]. Moreover, the antidepressant effect of HNK does not rely on direct modulation of NMDAR or other previously proposed KET targets [43–46]. Currently, the direct target of HNK action in brain remains elusive [20, 38, 45]. Moaddel et al. reported that KET metabolites norketamine, HNK, and dehydroxynorketamine, but not KET itself, inhibit acetylcholine-evoked currents in α7 nicotinic acetylcholine receptors (α7nAChRs) [47]. Preclinical studies also showed depression-like behavior upon disruption of α7nAChRs function and antidepressant-like properties of drugs that target α7nAChRs [48–51]. Nevertheless, the direct assessment of the role of α7nAChR in mediating the cellular effects of HNK has not yet been provided.

In the present study, we studied the significance of α7nAChR modulation by KET and HNK on cellular signaling. We focused on KET- and HNK-induced effects that elicit the cellular processes necessary for their rapid and sustained antidepressant action, namely neurotransmitter release and reconfiguration of gene expression. We demonstrated that both KET and HNK reduce the release competence of SVs, i.e. their availability for evoked fusion with the presynaptic membrane, which is necessary for presynaptic neurotransmitter release, and activate nuclear transcription factor CREB, which is necessary for antidepressant-induced gene expression reconfiguration. While α7nAChRs were required for the effect of HNK, the presynaptic effect of KET was evident also upon deletion or blockage of these receptors. Selective inhibition of GluN2B-containing NMDA receptors, a presumable target of KET antidepressant action, mimicked the presynaptic effect of KET. Thus, KET and HNK exert similar effects via modulating specifically glutamatergic and cholinergic neurotransmission.

## Materials and methods

### Animals

Dissociated rat primary cortical cultures were prepared from E18 Sprague Dawley (RjHan:SD) embryos obtained from Janvier Labs. Primary mouse neuronal cultures were prepared from B6(Cg)- *Chrna7*^<tm1.1Ehs>/Yakel^J mouse strain (further referred to as *Chrna7*^lox/lox^). This strain was purchased from Jackson laboratories (#026965) and backcrossed into the C57BL/6N background for more than five generations. Mice were bred in Franz-Penzoldt-Zentrum in Erlangen and experiments were carried out in accordance with local regulations and following the European Directive 2010/63/EU.

### Antibodies

Primary antibodies that were used in immunocytochemistry (ICC) and live antibody-uptake are as follows: rabbit antibody against synaptotagmin 1 (Syt1) (mouse cortical cultures, Oyster550-labelled, 1:100, #105103C3, Synaptic Systems), VGLUT1 (rat cortical cultures, 1:1000, #135303, Synaptic Systems), pSer133 of CREB (1:650, #9198 L, CST), mouse antibody against Syt1 (rat cortical cultures, Oyster550-labelled, 1:250, #105311C3, Synaptic Systems) and against VGLUT1 (mouse cultures, 1:250 #135311, Synaptic Systems). Primary antibodies that were used in western blotting are: guinea pig antibody against synapsin 1 (Syn1, 1:1000, #106104, Synaptic System), rabbit primary antibodies against pSer553Syn1 site (human and mouse pSer553 corresponds to pSer551 in rat Syn1, 1:1000, #ab32532, Abcam) and against pSer62Syn1 site (1:1000, #PA5-38336, Thermo Fisher). All the fluorescent secondary antibodies used in ICC were purchased from Jackson Immunoresearch and are as follows: anti-rabbit Alexa 488 (1:1000, #711545152), anti-mouse Cy5 (1:1000, #715175150) and anti-rabbit Cy3 (1:500 for mouse cultures, #711165152). For fluorescent detection of WB, IRDye 800CW donkey anti-guinea pig (#926-32411) and IRDye 680RD donkey anti-rabbit (# 926-6807) from Li-COR were used.

### Drugs and chemicals

Drugs used for treatment of rat and mouse cortical neurons were: R,S-ketamine hydrochloride (10 or 50 μM, #2753), 2R,6R-Hydroxynorketamine (R-HNK, 10 µM, #1430202-69-9) or 2S,6S-Hydroxynorketamine (S-HNK, 10 µM, #1430202-70-2) and Ro 25-6981 (1 µM, #1312991-76-6) all from Sigma, cis-6-hydroxynorketamine (10 or 50 μM, #5982) from Tocris, α-Bungarotoxin (50 nM, #11032794) from Calbiochem. D-(−)-2-Amino-5-phosphonopentanoic acid (APV, 50 µM, #0106) and 6-Cyano-7-nitroquinoxaline-2,3-dione disodium (CNQX, 10 µM, #0190) used in SV pool imaging were purchased from Tocris and Bafilomycin A1 (1 µM, #88899-55-2) from Invivogen. Bafilomycin A1 was dissolved in DMSO, remaining drugs were dissolved in water. Treatments were applied to individual wells in randomized manner and analyses were performed blinded.

### Primary rat cortical culture preparation

Primary rat cortical cultures were prepared as previously described [52]. Briefly, meninges-freed cortices were surgically isolated from rat E18 embryos, and then incubated with 0.25% trypsin (#15400054, Thermo Fisher Scientific) for 20 min at 37 °C. Next, tissues were mechanically triturated in presence of 0.1 mg/ml DNase I (#11284932001, Roche) to obtain a cell suspension which was filtered through 100 µm cell strainer and mixed with DMEM (#41966029, Thermo Fisher Scientific) based plating media with the following additives—10% (v:v) fetal calf serum (FCS, #S0015, Biochrom GmbH), 2 mM L-Glutamine (#25030024) and 1% (v:v) Antibiotic/Antimycotic (#15240062; both Thermo Fisher Scientific). Cells were seeded on 0.5 mg/ml Poly-L-Lysine (PLL, #P1524, Sigma-Aldrich) coated ø18 mm Menzel glass coverslips (#6311342, VWR international) in 12-well plates at the density of 150,000 cells/well and left to attach for 1 h at 37 °C. The plating media was then replaced with Neurobasal medium (#12348017) supplemented with 2% (v:v) B27 (#17504044), 0.8 mM GlutaMAX^TM^ (#35050-038) and 1% (v:v) Antibiotic/Antimycotic (#15240062; all from Thermo Fisher Scientific). Cultures were maintained in 5% CO_2_-containing humified incubator at 37 °C till the experimental date, and fed with fresh media once a week.

### *Chrna7*^lox/lox^ mice cortical culture preparation

Newborn (P0-P1) *Chrna7*^lox/lox^ mice were used for cortical culture preparation as we have described previously [52]. Briefly, cortical hemispheres were freed of soft cranial bones and meninges. Tissues were mechanically triturated and incubated at 37 °C for 10 min in a solution containing 0.127 U/ml papain (#LK003176, Worthington Biochemical Corporation), 1 mg/ml dispase II (#04942078001, Roche), 0.1 mg/ml DNase I (#LS002139, Worthington) and 12.4 mM MgSO_4_. The cell suspension was filtered through 70 μm nylon cell strainers, centrifuged at 120 g for 5 min and resuspended in Neurobasal A media (#12349-015) supplemented with 1% (v:v) GlutaMAX^TM^ (#35050-038), 1 mM sodium pyruvate (#11360-070), 1% (v:v) Antibiotic/Antimycotic and 2% (v:v) B27 (all from Thermo Fisher Scientific). 200,000 cells were plated as described for rat cultures and maintained in Neurobasal A media mix in 5% CO_2_ containing humified incubator at 37 °C till the experimental date, while 30% of media was replaced once per week.

### Lentiviral particle production and transduction

Active and inactive nuc-EGFP-cre recombinase (CRE and ΔCRE, respectively) constructs were a kind gift from Kaeser [53]. The ratio-sypHy-construct used for live imaging in this study has been reported previously [54–56]. Lentiviral particles were produced in HEK293T cells (ATCC) using FuGENE® HD (Promega) transfection agent following the manufacturer’s instructions. Briefly, 90% confluent HEK293T cells were transfected with FUGW-based CRE/ΔCRE/ratio:sypHy, psPAX2 (#12260, Addgene), and pVSVG (#8454, Addgene) pseudotyping vectors at 1.64:0.72:1.3 (in pmol) concentrations, respectively. Transfection media were replaced with supplemented Neurobasal A media 16 h after transfection. 24 h later lentiviral particles were collected and used for transduction of neurons from *Chrna7*^lox/lox^ mice or rats grown for 2 days in vitro (DIV2). The cre-mediated deletion of *Chrna7* was assessed by quantification of mRNA on cDNA from transduced neurons as described previously [52].

### Adeno-associated virus production

Adeno-associated viruses (AAVs) were used for expression of iGluSnFR for live-cell imaging of glutamate release. AAV2/1.hSynapsin1.SF-iGluSnFR.S72A vector was produced as previously described [57]. Briefly, HEK293T cells were transfected with the plasmids pAAV-DJ, pHelper, and pAAV-iGluSnFR in 1:1:1 ratio using the calcium phosphate precipitation method. After 3 days, cells were centrifuged and the supernatant was collected. The cell pellet was resuspended in 20 mM Tris-150mM NaCl buffer and cells were lysed by freeze-thawing cycles in liquid nitrogen. After centrifugation to remove cell debris, both supernatant stocks were mixed and incubated overnight at 4 °C with PEG-8000/0.5 M NaCl solution for precipitation of viral particles. Following this, viral particles were pelleted by centrifugation at 2500 *g* for 1 h and resuspended in TNE buffer (100 mM Tris, pH = 8.0, 150 mM NaCl, 20 mM EDTA). Aliquots were stored at −80 °C.

### Quantitative synaptotagmin 1 antibody uptake and immunocytochemistry (ICC)

Synaptotagmin 1 (Syt1) antibody (Ab) uptake was carried out in DIV18-21 rat cortical and DIV18 mouse cortical cultures as previously shown [52]. In order to monitor spontaneous activity-driven SV recycling cortical cultures were incubated for 20 min with Oyster 550 or Cy3-labeled antibody against lumenal domain of Syt1 (1:250 for rat neuronal cultures and 1:100 for mouse neuronal cultures) diluted in Tyrode´s buffer (TB; in mM: 119 NaCl, 2.5 KCl, 2 CaCl_2_, 2 MgCl_2_, 30 glucose, 25 HEPES; pH = 7.4). For evoked release of total recycling pool (TRP) of SVs, cortical cultures were labeled with Syt1 Ab in TB containing 50 mM KCl for 4 min at RT. Unbound antibody was then washed with TB and cultures were fixed with 4% (w:v) paraformaldehyde in PBS for 4 min at RT, blocked and permeabilized with PBS solution containing 10% (V:V) FCS and 0.1% (w:v) glycine and 0.3% (v:v) TritonX-100 for 40 min. Coverslips were incubated with primary antibodies overnight followed by secondary antibodies for 1 h at RT, both of which were diluted in PBS containing 3% (v:v) FCS. For Syt1 Ab-uptake in mouse neuronal cultures, an additional amplification of the fluorescent signal from Oyster 550-labeled primary antibody was done by incubation of coverslips with Cy3-labeled secondary antibody. Coverslips were then mounted on glass slides with home-made Mowiol or Fluroshield^TM^ (#F6182, Sigma-Aldrich) with or without DAPI. All experimental conditions investigated in each experiment were processed in parallel with identical antibodies, solutions, and other chemicals.

### Quantitative image acquisition and analysis

16-bit images of Syt1 Ab-uptake and quantitative ICC were acquired using motorized Ti Eclipse microscope equipped with 60X/NA1.2 water-immersion objective (CFI Plan Apo VC, Nikon), iXon EM + 885 EMCCD Andor camera (Andor Technology), and Omicron LedHUB illumination (Omicron-laserage Laserprodukte GmbH) and controlled by VisiView software (Visitron System GmbH). Identical camera and illumination settings were applied during image acquisition for coverslips of all experimental conditions imaged on the same day. An average of 10 visual fields were imaged from each coverslip. The synaptic immunofluorescence (IF) of Syt1 Ab-uptake was measured as described earlier [52]. Regions of interest were defined according to the VGLUT1 staining for synaptic IF quantifications. For quantification of nuclear pCREB133, images were acquired by a 20×/NA0.75 air objective. Mean IF was measured in spherical ROIs (7 × 6 µm) generated according to DAPI nuclear staining using ImageJ.

### Synapto-pHluorin (SypHy) imaging

48 h after seeding, rat cortical cultures were transduced with lentiviral particles expressing the ratio-sypHy-construct: a pH-sensitive GFP fused to the second intravesicular loop of the SV protein synaptophysin, as previously described [55]. Following drug and vehicle treatments at DIV18-21, cultured neurons were briefly washed with TB and placed in an electrical field stimulation chamber with platinum electrodes placed 1 cm apart (#RC-49MFSH without perfusion, Warner Instrument, Hamden, Connecticut, USA). Transduced neurons were identified by their RFP expression and live-imaging was performed at 30 ± 2 °C in presence of TB containing 10 μM CNQX, 50 μM APV and 1 μM bafilomycin A1 (vesicular V-type ATPase inhibitor) using the above mentioned epifluorescent microscope with an automated focus system (PFS) [52, 58]. The camera was controlled by VisiView software in 2 * 2 binning mode, for acquiring images at the frequency of 1 Hz. The protocol for imaging of SV pools was adapted from [59]. Briefly, after a baseline recording of 15 s, stimulation protocol comprising of 40 APs at 20 Hz (1 ms pulses of 100 mA) for triggering release of the readily releasable pool (RRP), followed by a strong stimulation of 900 APs at 20 Hz to release the TRP of SVs was applied using A385 stimulus isolator (World Precision Instruments) connected to a stimulus generator (#STG-4008, Multi-Channel Systems). Subsequent to stimulations, 60 mM NH_4_Cl was applied to achieve alkalization and unquenching of all SypHy-expressing vesicles. Visualization of synaptic puncta expressing SypHy was carried out using Cy3 filter-set (in mm: excitor 543/22 and emitter (593/40) and time lapse acquisition (at 1 Hz) of pHluorin response was done using GFP filter set (in mm: excitor 472/30 and emitter 520/35). Data were exported as stack files (.stk) containing 502 × 501 pixels of 16-bit monochromatic intensity values.

For data processing and analysis, .stk files were first imported in ImageJ and background was measured by placing circular ROIs with a diameter of 5 × 5 pixels on five independent regions in the image stack. Responding synaptic puncta were visualized by creating a maximal intensity z-projection of 45 frames during 900 AP stimulation. We observed minimal bleaching (≤0.02% per frame) from non-stimulated SypHy expressing neurons in our experiments. For detection of synaptic puncta, an automated SynQuant plugin, incorporated in ImageJ, was utilized to detect ROIs corresponding to synapses from the z-projection according to users application manual [60]. The SypHy response was read out in 150–200 detected ROIs per field of analysis. To obtain the fluorescence values for quantifications, the mean fluorescence intensities in ROIs were normalized by standard min to max feature scaling formula (F-Fmin)/(Fmax-Fmin), where Fmin corresponds to average intensity of baseline frames (12 frames corresponding to time interval between 3 and 15 s from the start of recording) and Fmax corresponds to the NH_4_Cl response. Only samples, where fluorescent intensity change was stable upon 40 and 900 AP stimulations and ≤80% of NH_4_Cl-evoked fluorescence were included for the analysis. The traces were plotted using the Time Series Analyzer V2.0 plugin in ImageJ. The relative fractions of RRP (measured from 10 frames corresponding to time interval between 40 and 50 s) and TRP (10 frames corresponding to time interval between 150 and 160 s) of SVs were calculated with respect to the maximum fluorescence reached upon NH_4_Cl alkalization Fmax.

### iGluSnFR imaging and analysis

Cortical cultures were transduced with AAVs for expression of iGluSnFR for live imaging of glutamate release on DIV7 and imaged after maturation on DIV18-21 following 30 min treatment with drugs. Same microscope and imaging/stimulation chamber were used as described above. Cells were imaged in presence of TB containing 10 µM CNQX and 50 µM APV at RT. After a baseline recording of 10 s, either three paired pulses (PP, 100 ms inter-pulse interval) spaced by 10 s followed by a burst stimulation of 10AP at 100 Hz or the burst stimulation only was delivered. Three-four recordings were acquired from different regions of one coverslip with an acquisition frequency of 60 Hz. Time-lapse series were processed in ImageJ using SynQuant plugin for synapse detection, similar to SypHy imaging. Average intensity z-projection of the frames recorded during burst stimulation were used to detect release sites. Samples that did not show fluorescent intensity change upon burst stimulation were excluded from analysis. To quantify the paired pulse ratio (PPR) the intensity of detected puncta (30–60 per movie) was measured throughout the time-lapse series and averaged to get single trace. Averaged baseline intensity (from 20 frames preceding each PP) was subtracted from the maximum fluorescence intensity recorded with the first pulse to calculate amplitude of first peak (P1). Similarly, fluorescence intensity recorded with second pulse was subtracted from averaged baseline intensity to calculate amplitude of second peak (P2). Ratio of second peak (P2) to first peak (P1) was calculated to obtain the final value of PPR. PPR traces are represented with ∆*F*/*F*0 feature normalization where *F*0 stands for baseline fluorescence. For quantifying response to the burst stimulation (10 AP at 100 Hz), mean fluorescence intensity values were first calculated using ∆*F*/*F*0 feature normalization and traces were obtained. Then, from these traces, average fluorescence intensity values of three consecutive frames during which maximum fluorescence intensity was reached upon were then quantified to obtain maximum amplitude of release.

### Quantitative western blot of primary cortical cultures

DIV18-21 cortical cultures were briefly washed with PBS and lysed with ice-cold RIPA buffer (150 mM NaCl, 1%(v/v) NP40, 0.5%(v/v) sodium deoxycholate, 0.1%(v/v) SDS); Additives - Complete Protease inhibitor Cocktail (Roche #04693116001), PhosSTOP^TM^ (Roche #04906837001). Lysates were collected and then centrifuged at 13,000 RPM, 4 °C for 10 min. The supernatant was collected to determine protein concentration using standard BCA assay. An equal amount of proteins (8–10 µg) were separated in 7.5% TGX^TM^ (Bio-Rad #4568023) stain-free gels fitted in a Bio-Rad 2-D electrophoresis chamber (Bio-Rad #1688005), and then blotted onto Bio-Rad low fluorescence PVDF membranes under semi-dry transfer conditions in Bio-Rad Turbotransfer^TM^ system. For immunodetection of phosphorylated and total Syn1, primary antibodies were diluted in 5% (v:v) BSA supplemented TBST. Fluorescent immunodetection was carried out in a LI-COR Odyssey^TM^ infrared scanner (at the signal emission spectrum of 680 nm or 800 nm, respectively). Immunofluorescence (IF) signals corresponding to phosphorylated Syn1 at sites Ser62 or Ser553 and total Syn1 were quantified in the same immunoblot from alternative channels using the detection and quantification software Image Studio V5.2 (LI-COR Biosciences). The immunoreactivity of all detected proteins was first normalized to TCE staining. Following this, ratio of immunodetected phosphorylated Syn1 Ser62 or Ser551 to respective total Syn1 was quantified. Samples from 4-5 independent primary neuronal cultures were used in these experiments.

### Statistics

Sample size for all experiments was set according to previous publications, where the comparable parameters were assessed using the same methodology [52, 61]. Statistical analyses were performed using Graphpad Prism 9 (GraphPad Software, San Diego, California, USA). All data points were normalized to respective controls and group size is indicated in the graphs. All data points for quantitative analyses are represented as mean ± SEM in text and depicted as scatter plot with bar format. Statistical analyses were performed using one-way ANOVA or two-way ANOVA as described in figure legends following statistical assumption of normality distribution. Assumptions of statistical tests (i.e., normal distribution and distribution of variance) were tested for each data set, and appropriate tests were then applied accordingly. Statistical significance is marked as * for *p* < 0.05, ** for *p* < 0.01, and *** for *p* < 0.001 in all plots.

## Results

### KET and HNK converge on regulation of SV recycling

**Fig. 1:**
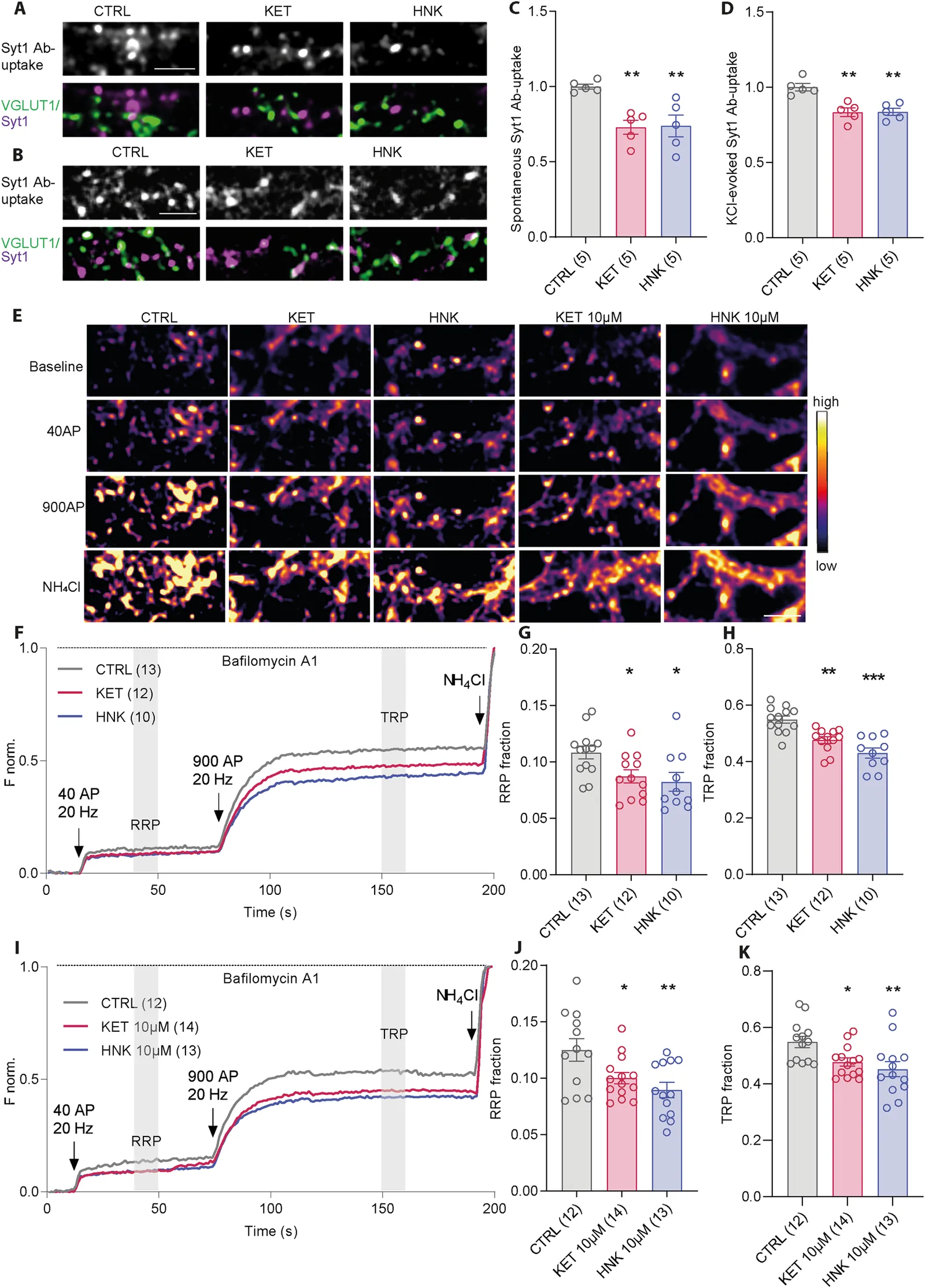
KET and HNK reduce the release competence of SVs. Representative images of network-activity driven (**A**) and KCL-induced depolarization driven (**B**) Syt1 Ab-uptake (magenta in overlay image) in DIV18-21 cortical neurons treated with vehicle (CTRL), KET or HNK for 30 min. Excitatory synapses are visualized by staining for VGLUT1 (green in overlay images). **C**, **D** Quantification of IF intensity experiments in (**A**) and (**B**). **E** Representative color gradient images show SypHy fluorescence at baseline and upon stimulation with 40 or 900 APs at 20 Hz and upon application of NH_4_Cl in DIV18-21 cortical neurons treated with CTRL and 50 or 10 µM KET or HNK. **F**, **I** Average traces show norm. SypHy fluorescence in cells treated with KET or HNK. Quantification of RRP (**G**, **J**) and TRP (**H**, **K**) fraction of SVs from traces in (**F** and **I**). Significance was assessed using one-way ANOVA with Dunnett’s multiple comparison’s test and depicted as ***p < 0.001, **p < 0.01, *p ≤ 0.05. Numbers in brackets correspond to the independently treated and analyzed coverslips used for staining or for live-cell imaging. Samples for each dataset originated from two to three independent culture preparations. Scale bar is 5 µm.

To compare effects of KET and its metabolite HNK on presynaptic function we treated mature cortical cultured neurons (DIV18-21) with 50 µM KET, 50 µM HNK or vehicle (CTRL). We used racemic mixtures of ketamine and cis-6 hydroxynorketamine, the most abundant HNK form found upon injection of KET in human. The acute effect of drugs on SV recycling was assessed 30 min upon treatment. An established synaptotagmin 1 antibody (Syt1 Ab) uptake assay [58] was used. The Syt1 Ab is fluorophore-coupled and recognizes lumenal domain of integral SV protein Syt1. When added to the media of cells, it labels specifically “active” synapses, where SVs fuse with plasma membrane during neurotransmitter release exposing the antibody epitope to the extracellular media, shortly prior to its internalization driven by compensatory endocytosis. In this way, Syt1 Ab-uptake immunofluorescence (IF) provides a measure of the SV exo-/endocytosis in living neurons at the level of individual synapses. Upon Syt1 Ab-uptake, the cells were fixed and labeled with antibody against vesicular glutamate transporter-1 (VGLUT1), an integral component of SVs, to identify excitatory synapses. First, we quantified the Syt1 Ab-uptake driven by endogenous activity of cultured neurons for 20 min upon application of Syt1 Ab. Neurons treated with both KET or HNK for 30 min prior to Syt1 Ab application showed significantly lower Syt1 Ab-uptake labeling at glutamatergic synapses compared to control (Fig. 1A, C; CTRL: 1 ± 0.01; KET: 0.0.73 ± 0.04; HNK: 0.74 ± 0.07).

Next, we tested whether the observed changes in spontaneous Syt1 Ab-uptake reflect the changes in release competence of SVs. To this end, we applied Syt1 Ab in medium containing 50 mM KCl for 4 min prior to cell washing and fixation. This brief depolarization evokes release of all release-competent vesicles [58]. Neurons pretreated for 30 min with either 50 µM KET or 50 µM HNK showed significantly reduced depolarization-induced Syt1 Ab-uptake compared to controls (Fig. 1B, D; Ctrl:1 ± 0.02; 0.83 ± 0.02; HNK: 0.84 ± 0.02). This is in accordance with the recent report showing the effect of KET and HNK on glutamate release in vitro and in vivo [17].

### KET and HNK converge on acute reduction in size of RRP and TRP of SVs

To further dissect the effects of KET and HNK on SV release, we performed live imaging experiment with cultured cortical neurons expressing a pH-sensitive probe called synaptophluorin-tdimer2 (SypHy) from a lentiviral expression vector [54]. SypHy contains a pH-sensitive GFP inserted within the lumenal domain of SV protein synaptophysin. Its fluorescence remains quenched in the intravesicular acidic pH = 5.5. It is transiently unquenched upon contact with neutral pH of the extracellular solution, and is quenched again upon endocytosis and vesicular re-acidification, thereby allowing fast real-time assessment of SV recycling at individual synapses. We utilized an established field stimulation protocol [59, 61] of 40 AP at 20 Hz to mobilize fusion of docked SVs corresponding to the readily releasable pool (RRP), followed by a train of 900 AP at 20 Hz in order to release SVs of the so-called recycling pool (RP). RRP and RP comprises of all release-competent SVs and signify the total recycling pool (TRP). Experiments were conducted in presence of vesicular proton pump blocker Bafilomycin A1, which prevents reacidification of endocytosed vesicles, allowing assessment of total releasable pool sizes. Ultimately, an ammonium chloride pulse was applied to alkalize and unquench the release-refractory SVs (corresponding to resting pool; RtP) (Fig. 1E, F).

Neurons treated with 50 µM KET or 50 µM HNK for 30 min prior to live-imaging experiments showed significantly lowered fraction of the RRP and TRP of SVs compared to vehicle-treated controls (Fig. 1G, H; RRP: CTRL: 0.10 ± 0.005; KET: 0.087 ± 0.006; HNK: 0.082 ± 0.008; TRP: CTRL: 0.54 ± 0.012; KET: 0.47 ± 0.012; HNK: 0.43 ± 0.017). We also tested the effects of lower concentration of KET and HNK, respectively, which are more relevant for their antidepressant property. We observed, similar reduction of RRP and TRP of SVs upon treatment with 10 µM KET or HNK, respectively (Fig. 1E, I, J, K; RRP: CTRL: 0.12 ± 0.01; KET: 0.10 ± 0.005; HNK: 0.08 ± 0.006; TRP: CTRL: 0.54 ± 0.02; KET: 0.47 ± 0.01; HNK: 0.45 ± 0.02). These results indicate that KET and HNK applied at 50 µM or 10 µM concentration rapidly inhibit SVs release by reduction of release-competent SVs in both the RRP and TRP.

**Fig. 2:**
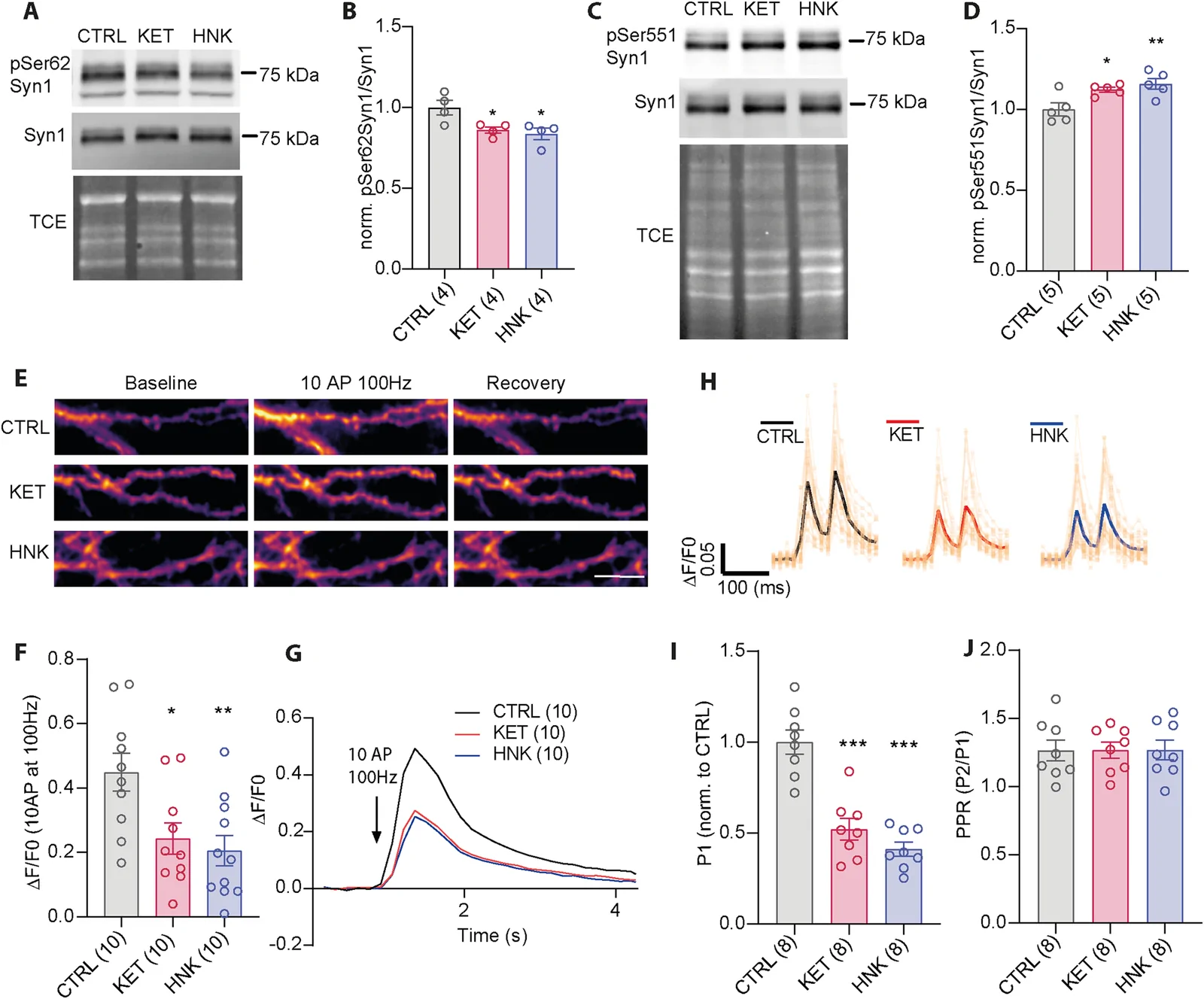
KET and HNK reduces SV release events and affect Syn1 phosphorylation. Representative immunoblot for Syn1 with pSer62Syn1 (**A**) or pSer551Syn1 (**C**) on lysates from CTRL and KET- or HNK-treated cortical neurons at DIV 18-21. Total protein staining was used as loading control. **B**, **D** Quantification of immunoblots is shown in (**A**, **C**) respectively. Values are expressed as pSer62 or pSer551Syn1/Syn1 ratio. **E** Representative images of iGluSnFR expressing neurons treated with vehicle, KET or HNK at baseline, upon stimulation with 10 AP at 100 Hz and upon recover. **F**, **G** Quantification of maximum response for all treatment conditions. **H** Averaged traces for responses to paired-pulse stimulation in cells treated with vehicle, KET and HNK. **I** Quantification of the response to first pulse (P1) **J** Quantification of PPR. Significance was tested using one-way ANOVA with Dunnett’s post-test and is indicated as ***p < 0.001, **p < 0.01, *p ≤ 0.05. Sample size (in brackets) corresponds to number of independently treated wells used for cell lysates (**B, D**) or coverslips used for imaging (**F, G, I, J**). Coverslips originated from at least two independent culture preparations. Scale bar is 5 µM.

One explanation for the KET- and HNK-induced reduction in release competence of SVs could be a change of phosphorylation in SV-associated phosphoprotein synapsin1 (Syn1). Syn1 oligomerize and interacts with F-actin in a phosphorylation-dependent manner, and thereby clusters SVs to the vicinity of presynaptic active zone, where neurotransmitters are released aligned towards the postsynaptic receptor apparatus [62]. Both KET and HNK were linked to activation of MAPK/ERK pathway, which is also known to mediate phosphorylation of Syn1 at Ser62 (pSer62Syn1) within its linker region [63–65]. To test this, we assessed phosphorylation of pSer62Syn1 in neurons treated for 30 min with 50 µM KET, 50 µM HNK or vehicle (CTRL) on Western blots using a phosphospecific antibody (Fig. 2A). Quantification revealed a significantly lower phosphorylation of Ser62 in treated cells compared to control (Fig. 2B; CTRL: 1.00 ± 0.04; KET: 0.86 ± 0.02; HNK: 0.83 ± 0.03). CDK5-driven phosphorylation site at Ser551 of Syn1 is known to reduce release competence of SVs, mobilizing their shift from TRP to RtP pool [66] and could, therefore, mediate reduction of TRP in KET- and HNK-treated neurons. The quantitative WB with specific antibody against pSer551Syn1 confirmed significantly increased phosphorylation at this site upon KET and HNK treatment (Fig. 2C, D; CTRL: 1.00 ± 0.03; KET: 1.12 ± 0.01; HNK: 1.15 ± 0.03). Taken together, identical effect of KET and HNK on reconfiguration of Syn1 phosphorylation represent a mechanism by which KET- and HNK-induced signaling converge on modulation of presynaptic function.

### HNK and KET reduce evoked neurotransmitter release without affecting presynaptic facilitation

Having established a reproducible effect of KET and its metabolite HNK on SV externalization, we tested effect of both drugs on evoked neurotransmitter release. Therefore, we employed an intensity-based glutamate-sensing fluorescent sensor SF-iGluSnFR.S72A (referred to as iGluSnFR) [57, 67]. Fluorescence of iGluSnFR increases rapidly, but transiently upon binding to glutamate and when expressed on the surface of cultured neurons it allows detection of electrically evoked glutamate transients at the level of individual synapses. First, we imaged glutamate release evoked by train of 10 AP delivered at 100 Hz. The amplitude of evoked iGluSnFR fluorescence was reduced by about 50% in cells treated with KET and HNK as compared to controls indicating reduced burst stimulation-induced glutamate release (Fig. 2E, F, G; CTRL: 0.44 ± 0.05; KET: 0.24 ± 0.04; HNK: 0.20 ± 0.04). Next, we applied stimulation with two pulses spaced by 100 ms, which allows measurement of presynaptic facilitation, a distinct form of short-term plasticity dependent, among others, on vesicular release probability [68]. The presynaptic facilitation expressed as paired-pulse ratio (PPR- fluorescence evoked by second pulse relative to the first pulse) remained unchanged in neurons treated with KET and HNK (Fig.2H, J; CTRL: 1.26 ± 0.07; KET: 1.26 ± 0.05; HNK: 1.27 ± 0.07). The response to the first stimulus was again lowered by 50% in neurons treated with KET or HNK (Fig. 2I; CTRL: 1.00 ± 0.06; KET: 0.52 ± 0.06; HNK: 0.41 ± 0.04). These data confirm that HNK and KET acutely reduce glutamatergic neurotransmission and are in line with reduced RRP that we observed upon drug application.

### R-HNK, but not its stereoisomer S-HNK, and selective inhibitor for GluN2B-containing NMDAR reduce SV release competence

**Fig. 3:**
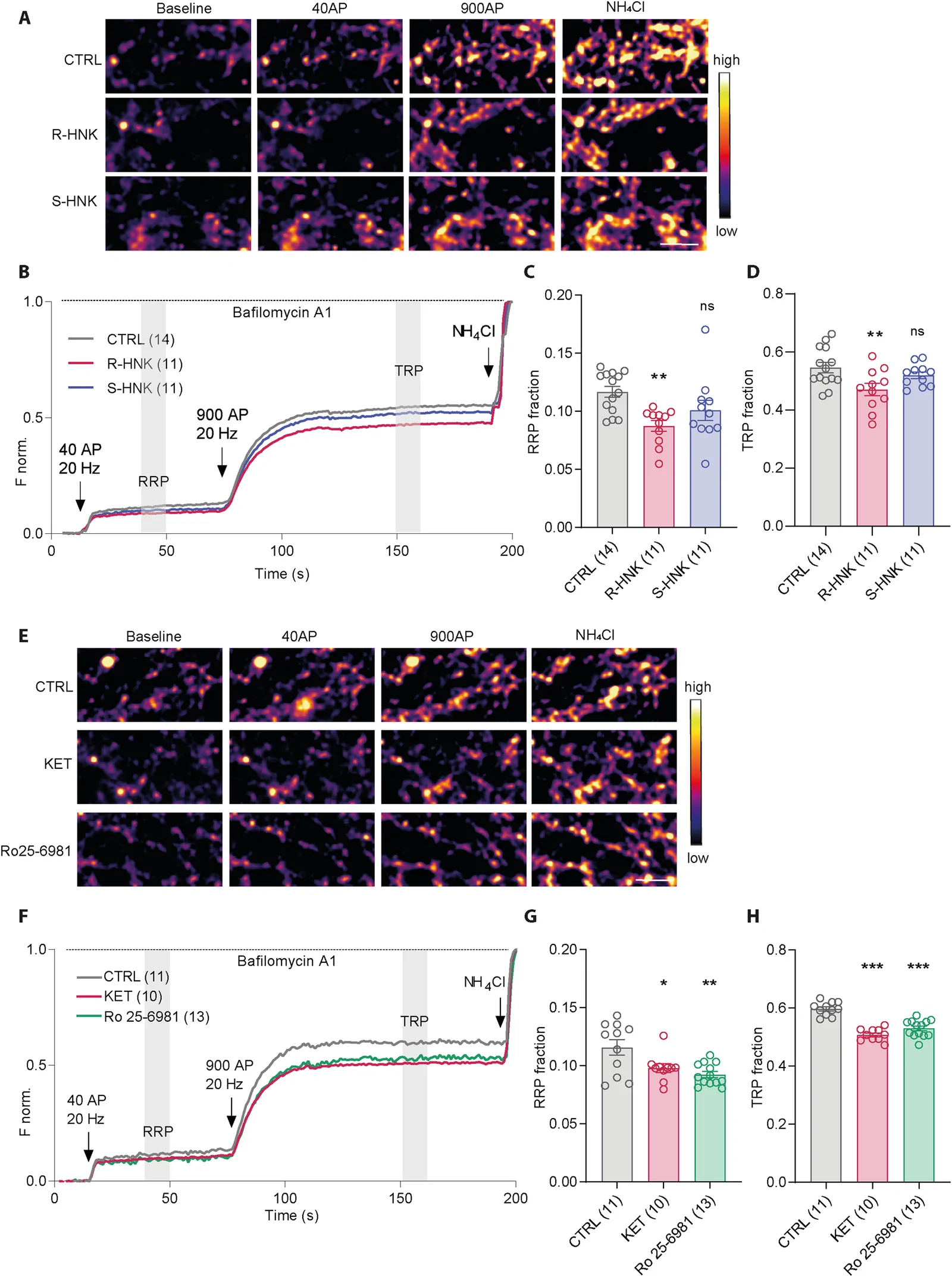
Effect of isomers of HNK and Ro25-6981, a GluN2B selective inhibitor, on SV release competence. **A**, **E** Representative color gradient images of SypHy fluorescence during rest and upon stimulation with 40 or 900 AP were acquired in DIV18-21 cortical neurons treated with R- or S-HNK (**A**) and with CTRL, KET or Ro25-6981 (**E)**. **B**, **F** Averaged traces show normalized SypHy fluorescence in neurons upon treatments in (**A**) and **(E**). Quantification of the RRP (**C**, **G**) and TRP (**D**, **H**) fractions from traces shown in (**A**) and (**E**). Significance was assessed using one-way ANOVA with Dunnett’s multiple comparison’s test and indicated as ***p < 0.001, **p < 0.01, *p ≤ 0.05. Sample size (in brackets) corresponds to the number of independently treated and analyzed coverslips obtained from two to three culture preparations. Scale bar is 5 µm.

The main HNK stereoisomers that are formed by metabolism of KET are 2 R,6R-HNK (R-HNK) and 2S,6S-HNK (S-HNK). Interestingly, R-HNK showed more potent antidepressant action compared to S-HNK in animal studies [20]. To assess, whether these stereoisomers have differing effect on presynaptic function, we compared their effect on release of SVs (Fig. 3A). We observed that RRP and TRP of SVs were significantly reduced upon treatment with 10 µM R-HNK for 30 min, while treatment with 10µM S-HNK had no effect (Fig. 3B–D; RRP: CTRL: 0.11 ± 004; R-HNK: 0.08 ± 0.004; S-HNK: 0.10 ± 0.008; TRP: CTRL: 0.54 ± 0.01; R-HNK: 0.47 ± 0.02; S-HNK: 0.51 ± 0.01).

Rapid-antidepressant effects of KET were linked to its ability to modulate among others GluN2B-containing NMDARs [35, 37, 39, 69]. Therefore, we investigated whether the GluN2B subunit-specific modulation also affects release competence of SVs. For this purpose, mature rat cortical neuronal cultures were treated with either vehicle, 50 µM KET or 1 µM Ro25-6981 for 30 min, prior to imaging SV pools (Fig. 3E). KET and Ro25-6981 had identical effect in significantly reducing the RRP and TRP of SVs compared to vehicle-treated cells (Fig. 3F–H); RRP:CTRL: 0.11 ± 0.006; KET: 0.09 ± 003; Ro25-6981: 0.09 ± 002; TRP: CTRL: 0.59 ± 0.006; KET: 0.50 ± 0.006; Ro25-6981: 0.53 ± 0.008). This argues for involvement of GluN2B-containing NMDAR in the observed regulation of functional pools of SVs at the synaptic ending.

### α7nAChR inhibitor bungarotoxin interferes with the effect of HNK on SV recycling whilst leaving KET-mediated effect unaffected

**Fig. 4:**
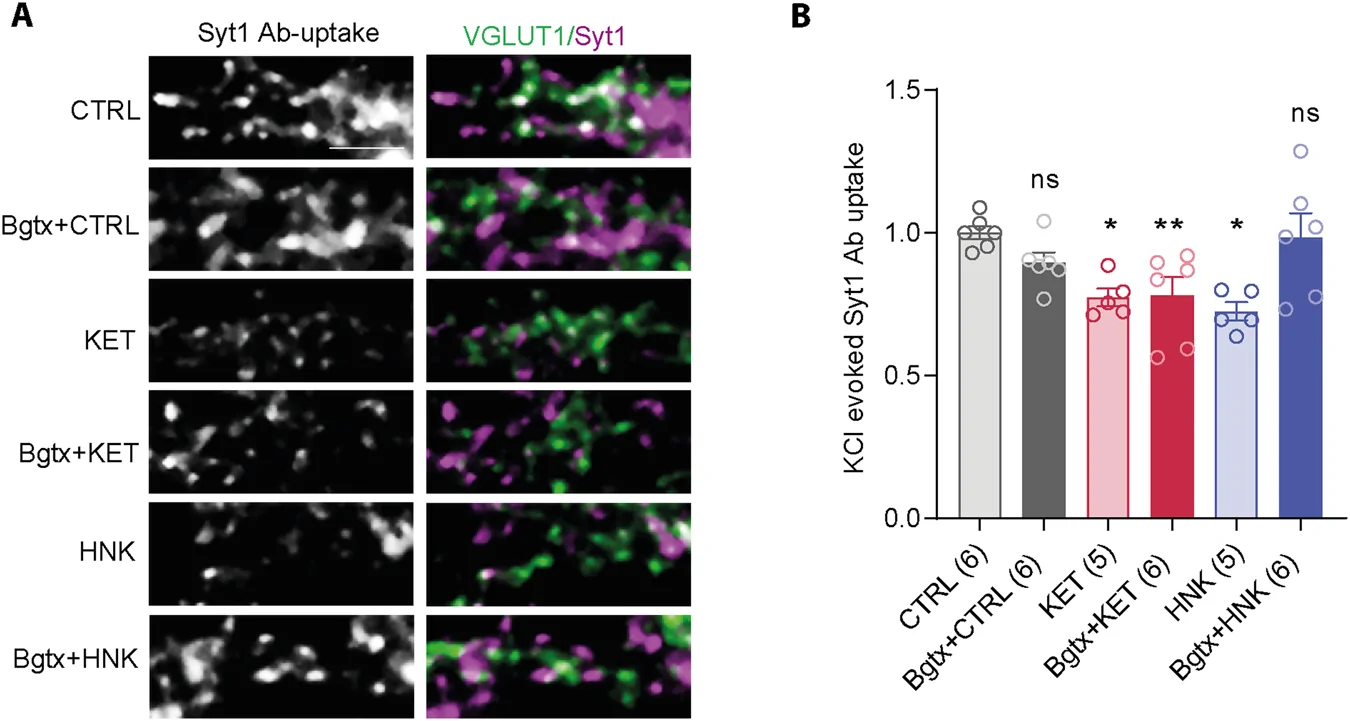
Pharmacological block of α7nAchRs prevents the effect of HNK on SV recycling leaving the effect of KET unaffected. **A** Representative image of Syt1 Ab-uptake IF (magenta) driven by KCl-induced depolarization in DIV21 primary cortical neurons that were treated with vehicle, KET or HNK in the presence or absence of the selective antagonist of α7nAChR bungarotoxin (Bgtx, 50 nM). Immunostaining for VGLUT1 (green) was used to visualize excitatory synapses. **B** Quantification of experiment in (**A**). Significance was assessed using two-way ANOVA with Holm-Šídák’s multiple comparison test and is denoted as ****p < 0.0001. Numbers in brackets indicate the number of coverslips analyzed from two to three culture preparations. Scalebar is 5 µM.

The receptor mediating the antidepressant effect of HNK is highly controversial. An older work showed a negative allosteric modulation of α7nAChRs by HNK at nM concentration [47]. To test the requirement of α7nAChRs for KET and HNK-dependent regulation of SVs, we pre-treated neurons with a selective α7nAChR antagonist α-bungarotoxin (50 nM, Bgtx) 30 min prior to HNK and KET treatments and the depolarization-induced Syt1 Ab-uptake assay. Two-way ANOVA confirmed the effect of KET and HNK treatment (*F*(2, 28) = 5413, *P* = 0.010) and interaction between these treatments and Bgtx pre-treatment (*F*(2, 28) = 6361, *P* = 0.0053), while no effect was seen for Bgtx pretreatment (*F*(1, 28) = 1530, *P* = 0.2264). The multiple testing confirmed that Bgtx pretreatment did not alter Syt1 Ab-uptake induced by chemical depolarization in neurons treated with vehicle (CTRL) (Fig. 4A, B; CTRL: 1 ± 0.02; Bgtx+CTRL: 0.89 ± 0.04) and had no effect on KET-induced reduction of Syt1 Ab-uptake at excitatory synapses (Fig. 4A, B; KET: 0.77 ± 0.03, Bgtx+KET: 0.78 ± 0.06). Importantly, Bgtx pretreatment fully abolished HNK-induced effect on Syt1 Ab-uptake which is in line with role of α7nAChR in mediating effect of HNK on SV release competence (Fig. 4A, B; HNK: 0.72 ± 0.02; Bgtx+HNK: 0.98 ± 0.08).

**Fig. 5:**
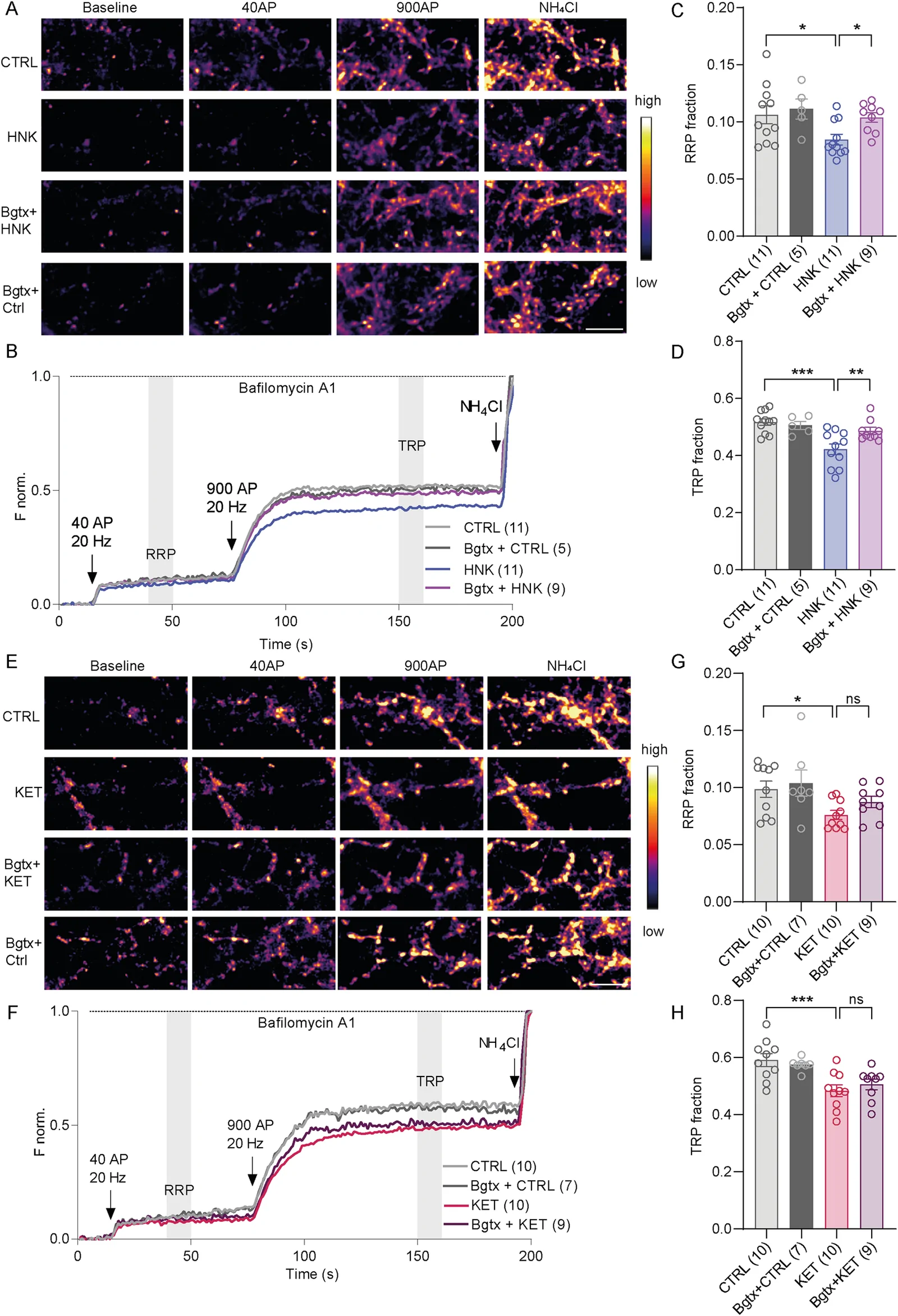
Pharmacological block of α7nAchRs prevents the effect of HNK treatment on SV release competence. Representative color gradient images of SypHy fluorescence at baseline, upon stimulation with 40 or 900 AP, and upon NH_4_Cl application in DIV18-21 cortical neurons treated with vehicle and HNK (**A**) or KET (**E**) with or without pretreatment with Bgtx. **B**, **F** Average traces from experiment in A or E are plotted following min to max feature normalization. Quantification of the RRP (**C**, **G**) and TRP (**D**, **H**) fractions from the experiment in (**A**) and (**E**), respectively. Significance was assessed by two-way ANOVA with Holm-Šídák’s multiple comparison test and marked ***p < 0.001; **p < 0.01; *p ≤ 0.05. Number of recordings each done on an independent coverslip derived from two to three independent culture preparations are indicated in brackets. Scale bar is 5 µm.

To confirm this important result with an independent technique, we performed time-lapse imaging with primary neurons expressing SypHy sensor in order to monitor SV pools as described above. Two-way ANOVA testing confirmed main effects of KET and HNK on both RRP and TRP (KET-RRP: *F*(1, 32) = 8.102. *P* = 0.0077), KET-TRP: *F*(1, 32) = 19.97, *P* < 0.0001; HNK-RRP: *F*(1, 33) = 5.098; *P* = 0.0307; and HNK-TRP: *F*(1, 32) = 12.23, *P* = 0.0014). Multiple post-testing revealed that pharmacological blockade of α7nAChR (Bgtx pretreatment) did not affect the RRP or TRP in vehicle-treated neurons, but it completely prevented HNK-mediated reduction of both RRP and TRP (Fig. 5A–D; RRP: CTRL: 0.11 ± 0.008; HNK: 0.08 ± 0.005; Bgtx+HNK: 0.10 ± 0.004; Bgtx+CTRL: 0.11 ± 0.01; TRP: CTRL: 0.50 ± 0.01; HNK: 0.41 ± 0.02; Bgtx+HNK: 0.48 ± 0.01; Bgtx+CTRL: 0.50 ± 0.01). Bgtx pretreatment did not significantly affect neither the KET-induced reduction in the TRP (Fig. 5E–H; TRP: CTRL: 0.59 ± 0.02; KET: 0.48 ± 0.02; Bgtx+KET: 0.50 ± 0.01; Bgtx+CTRL: 0.57 ± 0.01), nor the KET-induced decrease of RRP (Fig. 5E–H; RRP: CTRL: 0.09 ± 0.006; KET: 0.07 ± 0.003; Bgtx+KET: 0.08 ± 0.004; Bgtx+CTRL: 0.10 ± 0.011). This indicated that KET and HNK converge on regulation of the pool of release-competent SV acting via distinct signaling mechanisms: while effects of HNK require signaling via α7nAChRs, their pharmacological blockage did not affect KET-induced changes.

### Genetic ablation of α7nAChR occludes the effect of HNK on SV recycling without affecting KET effect

**Fig. 6:**
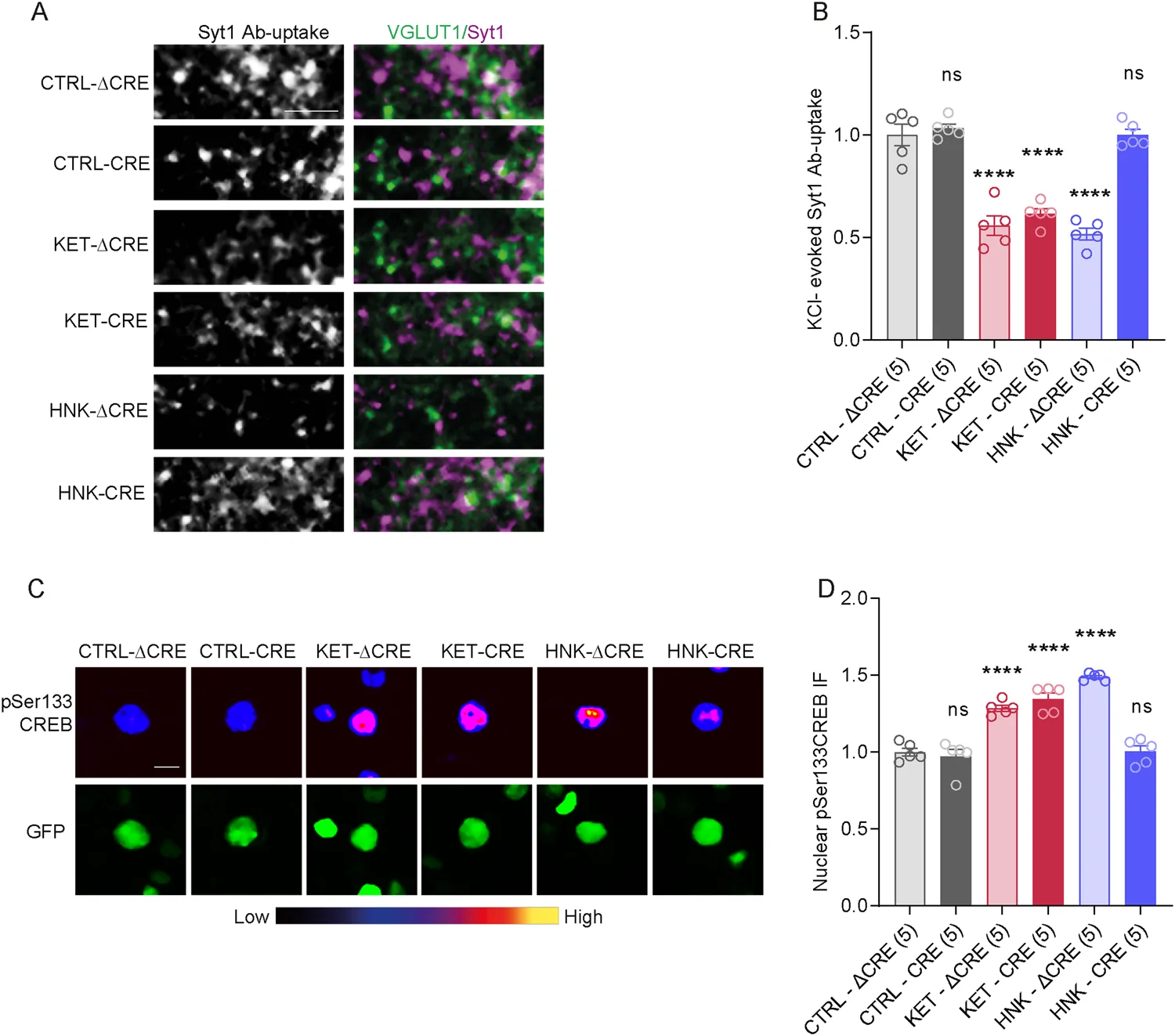
Genetic ablation of *Chrna7* prevents HNK-mediated synaptic and nuclear effects leaving KET-mediated effects unaffected. **A** Representative images showing KCl-induced depolarization-driven Syt1 Ab-uptake IF (magenta) in VGLUT1-positive (green) synapses in DIV18 cortical neurons treated with vehicle, KET or HNK. The cultures were prepared from Chrna7 floxed animals and infected with a virus that expressed control (∆CRE) and or active cre recombinase (CRE) leading to a genetic deletion of α7nAchRs. **B** Quantification of Syt1 Ab-uptake from experiment in (**A**). **C** Representative pseudocolour-gradient images of pSer133CREB nuclear IF in neurons treated with vehicle, KET and HNK, green marks expression of ∆CRE and CRE in cell nuclei. **D** Quantification of pSer133CREB nuclear IF from **C**. Significance was assessed using two-way ANOVA with Holm-Šídák’s multiple comparison test and depicted as ****p < 0.0001. Sample size (in bracket) corresponds to the number of independently treated coverslips. Cells originated from two to three independent culture preparations. Scale bar is 5 µm in (**A**) and 10 µm in (**C**).

To further substantiate the specific involvement of α7nAChRs in HNK-dependent signaling at presynapse, we investigated the effects of KET and HNK in primary neurons upon genetic deletion of the pore-forming subunit of α7nAChR, *Chrna7*. Primary cultures were prepared from *Chrna*7^lox/lox^ mice, which have loxP sites flanking the exon 4 of *Chrna7*. On DIV2, these neurons were transduced with lentiviral particles expressing EGFP targeted to the nucleus and fused to cre recombinase (CRE) or with particles expressing only nuclear EGFP (∆CRE) as a control. On DIV18, below-threshold expression of *Chrna7* mRNA was detected in CRE expressing cells. On DIV18, neurons were treated with KET, HNK, or vehicle (CTRL), and Syt1 Ab-uptake assay was done to monitor chemical depolarization-induced release of TRP at excitatory synapses (Fig. 6A). Two-way ANOVA revealed significant effect for KET/HNK treatment (*F* (2, 24) = 72.58, *P* < 0,0001) and *Chrna7* knock out (*F*(1, 24) = 42.52, *P* < 0,0001) and confirmed interaction of these two variables (*F*(2, 24) = 24.96, *P* < 0,0001). Multiple testing confirmed that both, KET and HNK, significantly reduced Syt1 Ab-uptake after 30 min in ∆CRE-infected neurons with intact *Chrna7* (Fig. 6A, B; CTRL-∆CRE:1 ± 0.05; KET-∆CRE: 0.55 ± 0.04; HNK-∆CRE: 0.51 ± 0.02). Contrarily, in CRE-infected neuronal cultures KET treatment elicited a decline in Syt1 Ab-uptake compared to CTRL, but HNK had no effect (Fig. 6A, B; CTRL-CRE:1 ± 0.05; KET-CRE:0.61 ± 0.02; HNK-CRE:1 ± 0.02). These data provide further independent confirmation that in contrast to KET, HNK modulates release competence of SVs in a α7nAChR-dependent manner.

### HNK but not KET require functional *Chrna7* expression to activate CREB

Transcriptional factor cAMP response element-binding protein (CREB) is a major regulator of gene expression implicated in depression and in antidepressant actions of KET and HNK in human and animal models [70]. α7nAchR was linked to the regulation of intracellular cAMP in multiple cell types, including neurons [71]. To investigate whether these receptors are also important for the regulation of nuclear CREB activity by KET and HNK, we measured nuclear accumulation of phosphorylated CREB at Ser133 (pSer133CREB) in *Chrna*7^lox/lox^ neurons expressing CRE or ∆CRE upon treatment with vehicle, KET or HNK. pSer133CREB was visualized by immunostaining using a phosphospecific antibody (Fig. 6C). Two-way ANOVA revealed significant effect for KET/HNK treatment (*F* (2, 24) = 57.81, *P* < 0.0001) and *Chrna7* knock out (*F*(1, 24) = 32.52, *P* < 0.0001) and confirmed interaction of these two variables (*F*(1, 24) = 41.03, *P* < 0,0001). In neurons infected with ∆CRE particles, where expression of *Chrna7* remained intact, we observed increased nuclear pSer133CREB IF 30 min after treatment with both KET and HNK as compared to vehicle-treated cells (Fig. 6C, D; CTRL-∆CRE:1.0 ± 0.02; KET-∆CRE:1.28 ± 0.02; HNK-∆CRE:1.48 ± 0.01). This is in line with previously proposed role of CREB signaling in antidepressant effects of both compounds [44]. In cells where infection with CRE particles abolished *Chrna7* expression, the KET-induced CREB activation remained unchanged, but effect of HNK was completely abolished (Fig. 6C, D; CTRL-CRE:0.97 ± 0.04; KET-CRE:1.34 ± 0.03; HNK-CRE: 1.0 ± 0.03). These results showed that expression of *Chrna*7 is specifically required for activation of transcriptional factor CREB by HNK, but not for KET-dependent CREB activation.

## Discussion

We have shown that KET and its metabolite HNK converge on similar presynaptic regulations. They rapidly reduce the number of the releasable SVs and thus dampen neurotransmission. Specifically, 30 min long treatment with KET and HNK led to a reduction in number of SVs in the RRP, which determines presynaptic release efficiency during the evoked neurotransmission, and in TRP, which contributes to the SV release evoked by repetitive stimulations [72]. Glutamate imaging confirmed that lower evoked neurotransmission upon single stimuli as well as upon trains of stimuli with unchanged paired-pulse facilitation, which is in line with drug-induced reduction of releasable SVs. These data provide a mechanism for earlier reported effects of acute systemic application of KET, Ro25-6981 or HNK leading to a significant decline in glutamate release induced by prolonged depolarization in mouse PFC and subiculum, as well as in vitro [16, 17]. The decline in the size of RRP, comprising of the SVs docked at their release sites, is in good agreement with previously published biochemical data. Systemic administration of subanaesthetic dose of KET rapidly inhibited autophosphorylation of Thr^286^ of α calmodulin-dependent kinase II (pThr^286^αCaMKII), which is necessary for its interaction with the SNARE component syntaxin1A and consecutive SNARE complex assembly [40]. Reduced autophosphorylation of pThr^286^αCaMKII was detected also upon an application of HNK [17] supporting converging inhibitory effect of KET and its metabolite HNK on SNARE formation resulting in reduction of size of RRP of SVs, as our study now reveals directly. Synapsins are the main molecular determinant of release competence of SVs, since they regulate SV clustering within the presynaptic compartment and mobilization, for trafficking towards release sites [62]. We investigated 1) MAPK-dependent phosphorylation of Syn1 on Ser62, which is known to increase RRP and recruitment of vesicles to recycling pool, and 2) CDK5-dependent phosphorylation of Syn1 at Ser551, known to shift SVs from TRP to RtP by increasing their clustering and restricting their mobilization [66, 73–76]. We have detected converging effect of KET and HNK on phosphorylation of Syn1 [62], the MAPK-dependent pSer62Syn1 was significantly reduced and the CDK5-dependent pSer551Syn1 was increased in KET and HNK-treated neurons, which is in line with the observed KET- and HNK-induced reduction of RRP and TRP. Thus, KET and HNK induce cellular signaling that modulate presynaptic function via modulation of common cellular signaling cascades. Moreover, these data indicate that analogous regulation of presynaptic function by KET and its metabolite HNK potentially represent a common important physiological step towards their antidepressant action.

Despite their common antidepressant action, KET and HNK differ in multiple biological actions. KET has pronounced adverse effects including dissociation, psychotomimetic effects, cognitive impairment and abuse liability, which were hitherto not reported for HNK in preclinical studies [20, 22, 26, 77]. There is a broad consensus about importance of NMDA receptor for antidepressant and adverse effect of KET [78]. Multiple studies confirmed that antidepressant-like effect of HNK does not rely on NMDAR inhibition [20, 45], but the molecular target of HNK was hitherto unclear [46]. In our experiments, we demonstrated that HNK-induced modulation of presynaptic function, as well as activation of nuclear transcriptional factor CREB was dependent on functional expression of α7nAChRs. Testing of HNK stereoisomers revealed that this effect was more pronounced for R-HNK, while it was not significant for S-HNK. An acute inhibition of these receptors with selective blocker Bgtx, as well as their genetic ablation completely abolished the HNK induced effect. However, the same manipulations did not affect KET-induced regulation at synaptic ending and nucleus revealing that KET and HNK act on different molecular targets.

How can KET and HNK elicit similar systemic and cellular effects acting on different membrane receptors? Both GluN2B-containing NMDARs and α7nAChRs are ion channels conducting Ca^2+^ currents, which might explain the convergent cellular effect of their modulation by KET and HNK, respectively. The involvement of Ca^2+^ as the secondary messenger in HNK-dependent presynaptic regulations is in line with the recent data showing that positive allosteric modulation of presynaptic α7nAChRs by physiological Aβ metabolites relies on activation of the Ca^2+^-dependent phosphatase calcineurin, resulting in an increased mobilization of SVs during repetitive stimulation and promoting presynaptic strengthening [52]. Calcineurin is a known downstream effector of presynaptic NMDARs involved in NMDAR-induced increase in presynaptic short-term plasticity at glutamatergic synapses of cortex or hippocampus [79–81]. Moreover, the cellular signaling induced by both compounds including RAS-MAPK, PI3K/Akt/mTOR activation and local translation are regulated by Ca^2+^ influx in neurons [17, 20, 38, 82]. We did not directly address the role of calcium signaling in this work, but it will be interesting to approach this in future studies.

The identification of α7nAChR as a molecular target of HNK action is the most striking finding of this study. Nicotinic AChRs have been traditionally linked to MDD [83, 84]. Early observations of depressive symptoms induced by infusion of physostigmine, a nonspecific inhibitor of acetylcholine esterase, in human probands and animal models as well as comorbidity between nicotine-dependence and MDD supported this hypothesis [85–89]. Increased CHRNA7 copy numbers were observed in adolescent and young adult MDD patients, but decreased levels of α7nAChR were detected in patients with unipolar depression [90, 91]. Previous imaging studies described slightly elevated choline concentration in prefrontal lobe on MDD patients as well as normalization of abnormal cortico-striatal connectivity in MDD patients upon nicotine administration [92, 93]. However, more advanced analysis should confirm these data. In mice, manipulation of α7nAChR leads to depression-like behavioral features and administration of their antagonists or modulators had antidepressant-like effects [48, 50, 51, 94, 95]. Multiple clinical trials with nicotine showed significant but unfortunately only transient improvements in depressive symptoms, which was attributed to its role as agonist of nAChR [96]. However, a positive allosteric modulator of α7nAChR, JNJ-39393406, failed to improve depressive symptoms in patients [97]. Mecamylamine, a nicotinic antagonist acting via α7 and β2 subunits, improved depression symptoms when used as adjunct to ongoing antidepressant therapy in a smaller patient cohort, but this effect was not confirmed in following study with larger cohort [51, 98, 99]. Future clinical studies with negative allosteric modulators, having effect on α7nAChR similar to that of HNK, or with HNK itself should test their potential therapeutic potential. It is conceivable that the therapeutic effect of KET in MDD arises from a consecutive action of rapidly-metabolized ketamine and its metabolite HNK on glutamatergic and cholinergic neurotransmission. In this way, activity of distinct cell populations throughout the brain could be modulated across the time. This finding might stimulate exploration of new combined medication for MDD.

## Data Availability

This study includes no data deposited in public repositories.
